# Delayed Recognition of Thoracic and Lumbar Vertebral Compression Fractures in Minor Accident Cases

**DOI:** 10.7759/cureus.1050

**Published:** 2017-02-23

**Authors:** Jesse Hatgis, Michelle Granville, Robert E Jacobson

**Affiliations:** 1 Larkin Hospital, Nova Southeastern University School of Osteopathic Medicine; 2 Miami Neurosurgical Center, University of Miami Hospital

**Keywords:** vertebral compression fracture, motor vehicle accident, accident clinic, compression fracture

## Abstract

Osteoporotic vertebral compression fractures (VCFs) in the elderly are commonly diagnosed after a minor fall or trauma; however, the majority of these patients have either been previously evaluated for osteoporosis or are already under some form of medical treatment for osteoporosis at the time of the fall. Although accidents are a known cause of VCFs, these fractures are too often undiagnosed. In reviewing a group of patients seen after minor falls or automobile accidents who were complaining of general spine pain, we found a smaller subgroup with previously undiagnosed VCFs. These fractures were also the initial signs of a previously unrecognized osteoporotic process. Initial diagnosis, treatment, and therapy were usually focused on other spinal segments (i.e. mainly the lumbar spine) until both the VCF and the osteoporosis were identified. The purpose of this report is to raise awareness and discuss the steps for improved diagnosis of osteoporotic VCFs.

A retrospective analysis was conducted on a large group of patients from one pain/accident clinic in a 24 month period. These patients were diagnosed with VCFs subsequent to the initial evaluation due to either persistent pain after conservative therapy or complaints of pain beyond the original injured area (i.e. typically the lumbar spine). At this point, a more detailed history was taken, including any past treatment for osteoporosis, or previous falls or injury to exclude the possibility of pre-existing fractures. A more focused examination of the painful area was completed, consisting of percussion at the fracture site identified on magnetic resonance imaging (MRI) or computed tomography (CT) scan. If possible, a bone scan was ordered to separate acute and subacute traumatic fractures from old/chronic fractures. Additionally, we surveyed two other similar pain/accident clinics who saw a comparable number and population of patients diagnosed with VCFs within a 24 month period to make a comparison of the number of VCFs they identified.

Ten out of approximately 2700 patients seen over a 24 month period sustained acute thoracic or lumbar VCFs during a minor accident and were not previously diagnosed with osteoporosis. Since approximately 30% of the 2,700 patients had new accidents, 10 out of 800 new patients (1.25%) were found to have VCFs without a known history of osteoporosis. Two other surveyed pain/accident, clinics saw a similar number and population of patients in the same time period; however, each only diagnosed two or three VCFs while examining a similar number of patients in the clinic. In these two other clinics, a much lower percentage (0.3%) of patients were diagnosed with new VCFs.

Awareness of the possibility of osteoporotic VCFs is the first step in detecting them. This study reveals the presence of a small but real risk of overlooking osteoporotic VCFs in minor trauma cases. When necessary, repeat or obtain better quality imaging in spinal segments affected by persistent pain. The thoracolumbar junction (i.e. T12 & L1 vertebrae) is especially at risk for sustaining VCFs. The delayed recognition of these VCFs and the patient's underlying osteoporosis after minor accident cases could present a major problem, as the critical time for patients to receive the proper medical or surgical treatments responsible for correcting and preventing further spinal deformity and pain has been reduced.

## Introduction

Osteoporotic vertebral compression fractures (VCFs) in elderly patients are well recognized and often acutely symptomatic [1]. VCFs are usually diagnosed after a fall or lifting accident [1]. The majority of these patients who sustained VCFs have previously undergone bone density testing and treatment for osteoporosis. In contrast, there is a small group of patients who were previously undiagnosed with osteoporosis in which the sustained VCF was the initial sign of this disease process, subsequently leading to further diagnostic testing and treatment.

Although accidents are a known cause of VCFs they are frequently undiagnosed [2-3]. We have identified a subgroup of patients who were seen after minor falls or automobile accidents complaining of multiregional spine pain. Despite not being previously diagnosed with osteoporosis, they were found on diagnostic imaging to have fresh VCFs related to the accident. These were initially overlooked by other clinics and led to a delay in VCF diagnosis and treatment since often treatment and therapy were focused on other areas of the spine. The goal of this report is to raise awareness and discuss the steps for improved diagnosis of osteoporotic VCFs. Informed consent was obtained from the patient for this study.

## Materials and methods

We performed a retrospective analysis on a large group of patients from one pain/accident clinic in a 24 month period. The patients of interest had been diagnosed with vertebral compression fractures after initial evaluation due to either persistent pain after conservative therapy or treatment of pain beyond the original injured area (i.e. typically in the lumbar spine). At this point, a more detailed history of any past treatment for osteoporosis and previous falls or injury to exclude pre-existing fractures was taken. A localized examination of the painful area was completed, consisting of percussion for localized pain at the fracture site. If possible, a bone scan was ordered to separate old/chronic fractures from the more acute and subacute traumatic fractures. Two patients were significantly younger in age and sustained a severe acute trauma. Their vertebral compression fractures were diagnosed either on initial examination at a hospital or during an initial clinic evaluation on radiographs. These cases were not included in the final percentage of patients who sustained osteoporotic VCFs. We also surveyed two other similar pain/accident clinics that saw a similar number and population of patients diagnosed with VCFs within a 24 month period.

## Results

Ten out of approximately 2700 patients fit our inclusion criteria of sustaining acute thoracic or lumbar VCFs during a minor accident and not having been previously diagnosed with osteoporosis. There were originally 12 patients identified with VCFs. The group of 12 were composed of eight females and four males. Two of the male patients had acutely diagnosed VCFs at the time of the initial accident, were significantly younger in age and had sustained initial severe trauma, so their data was not included in the final analysis. Approximately 30% of the 2,700 patients evaluated had new accidents, thus 10 out of 800 new patients (1.25%) were found to have VCFs without a known history of osteoporosis. All of the remaining 10 patients reviewed were not originally diagnosed with either VCFs or osteoporosis at the time of the initial accident or at the initial clinic evaluation. The two other surveyed pain/accident clinics that saw a similar number and population of patients in the same time period each only diagnosed two or three VCFs. This results in a much lower percentage (approximately 0.3%) of patients who sustained new VCFs in minor accidents. Two patients sustained multi-level VCFs whereas eight sustained only a single-level VCF. 6/8 VCFs were at the thoracolumbar junction of T12 or L1 (Table 1).

**Table 1:** Vertebral compression fracture levels

Patient	Age	Sex	Fracture Level(s)
A	72	F	T3 & T4​
B	68	F	T6, T8, & T11 (figure 1)
C	69	F	T9
D	58	M	T12
E	76	F	T12
F	79	F	T12
G	75	F	L1 (figure 2)
H	89	M	L1
I	61	F	L1
J	63	F	L2 (figure 3)

For radiographic examples, please refer to Figure 1, Figure 2, and Figure 3 below:

### Example #1:

**Figure 1:**
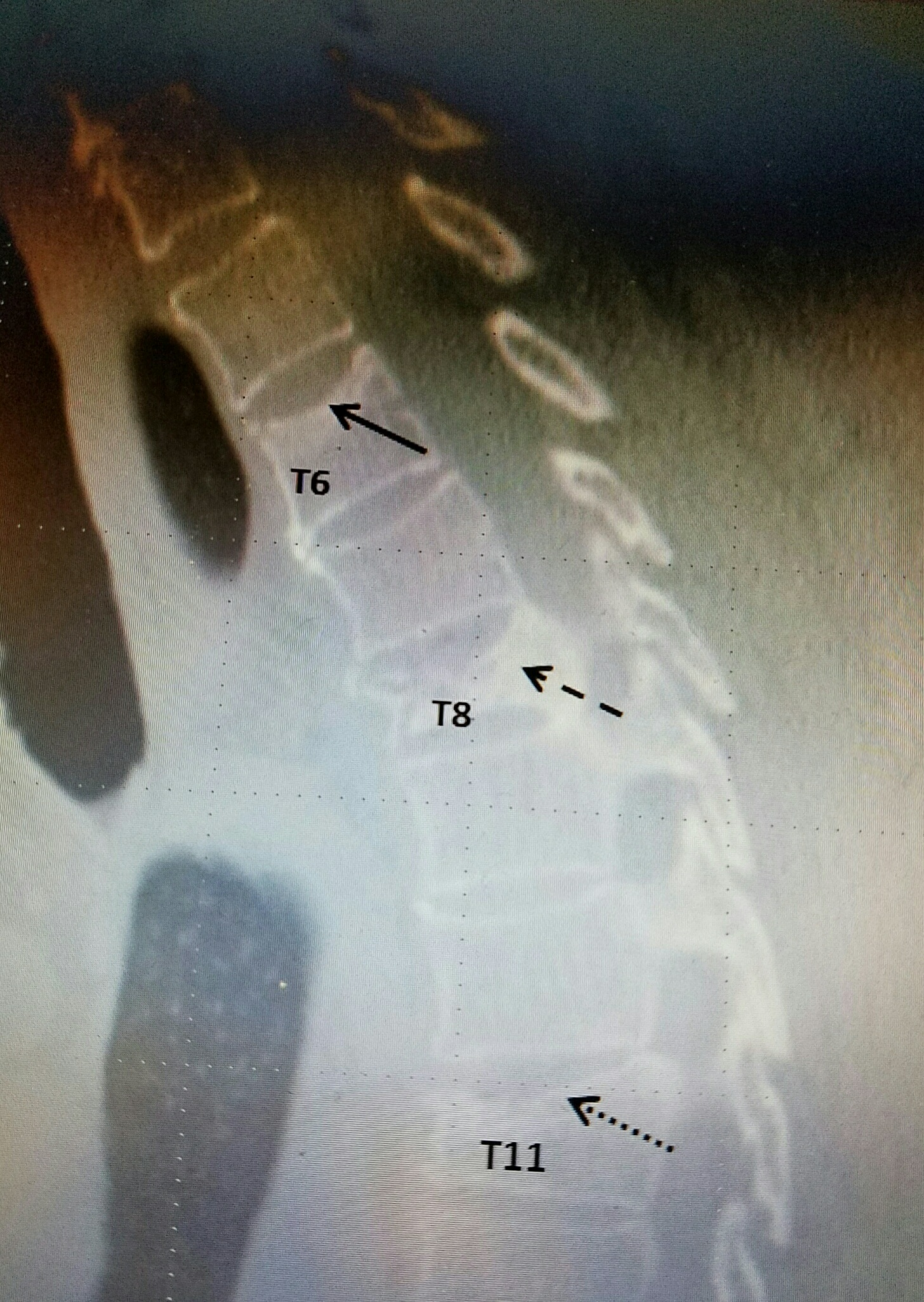
Thoracic spine CT scan sagittal view in a patient who complained of middle to lower back pain. Three vertebral compression fractures are shown at different progressive stages: T6 superior endplate fracture with minimal height loss (solid arrow), T11 compression fracture with moderate height loss (dotted arrow), and T8 compression fracture with severe height loss (dashed arrow)

### Example #2:

**Figure 2:**
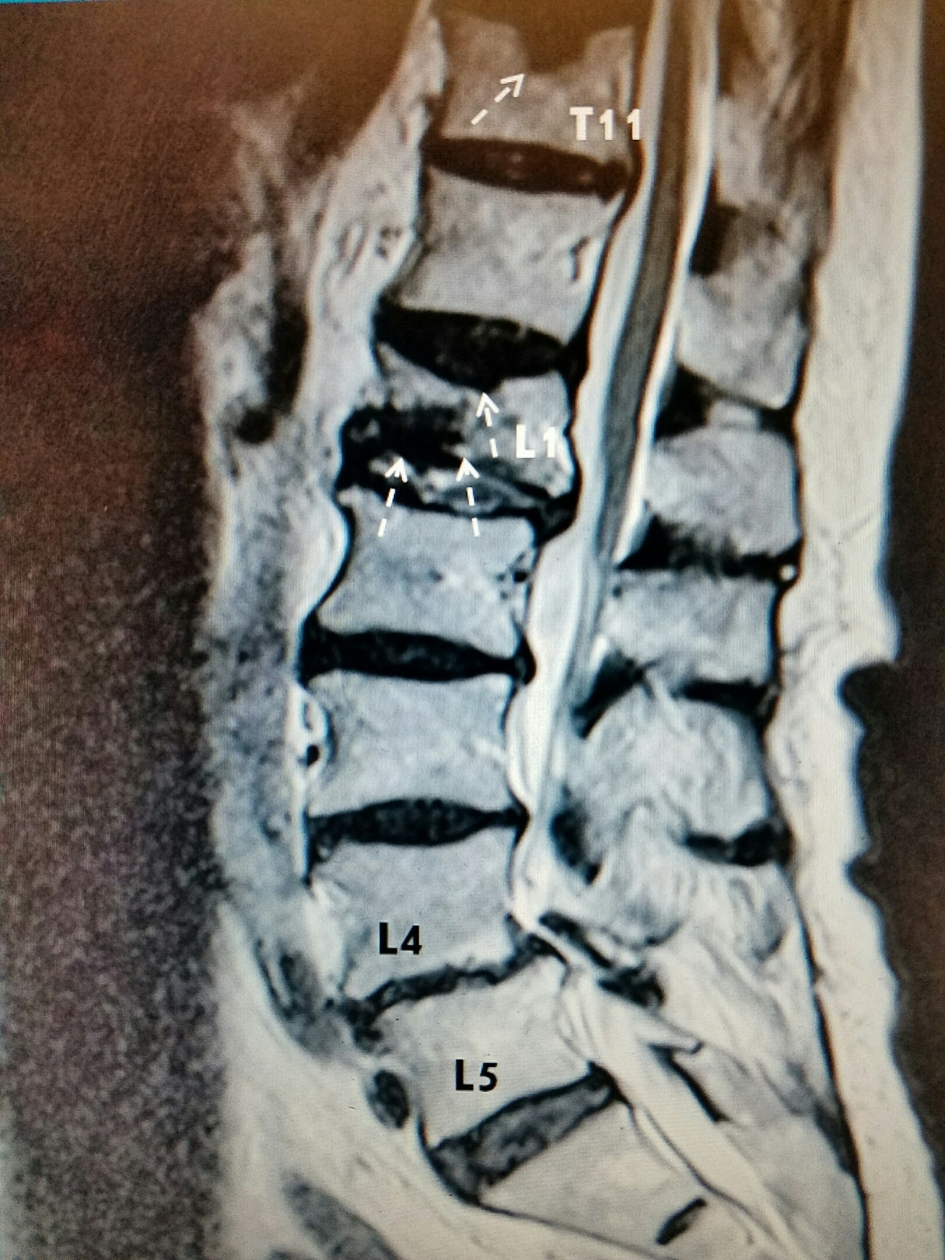
Lumbar spine MRI sagittal view of a patient with lower back pain. A T11 schmorl node with a chronic superior endplate fracture (single dashed arrow), acute L1 superior and inferior endplate fractures with vacuum phenomenon (three dashed arrows), and L4-L5 grade I anterolisthesis with multilevel spinal stenosis are appreciated

### Example #3:

**Figure 3:**
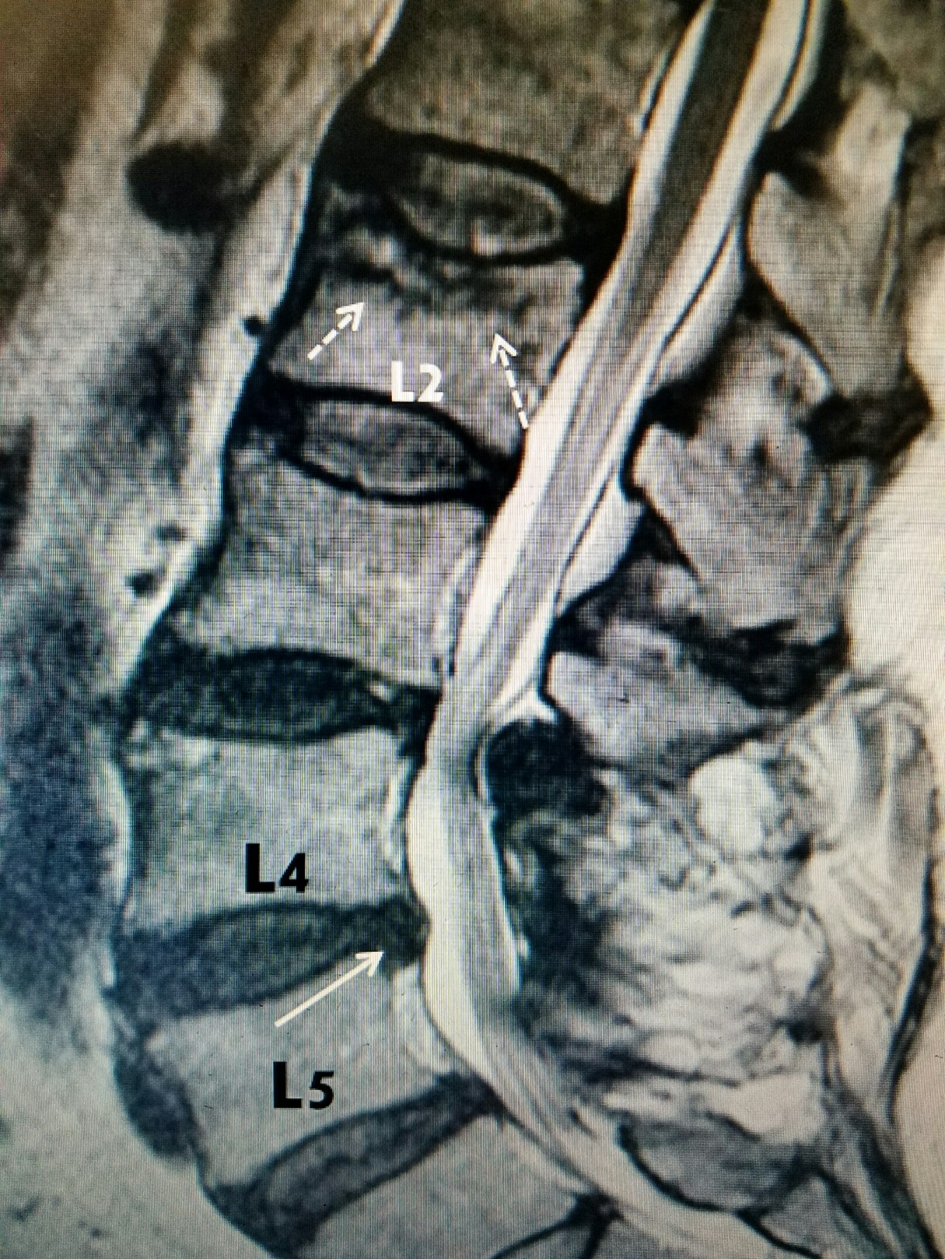
Lumbar spine MRI sagittal view in a patient with lower back pain demonstrating an acute L2 superior endplate fracture into the trabecular bone with edematous changes (dashed arrows) plus a small L4-L5 disc herniation (solid white arrow) with posterior facet hypertrophy at L3-L4

## Discussion

Although at first glimpse the resulted small percentages do not appear to be of great clinical meaning, these numbers extrapolated over time and hundreds of patients do represent a meaningful difference. Each of the two other clinics (seeing a mix of the same type and age group of patients) only diagnosed one-fifth (21%) of the number of patients with vertebral compression fractures that the reference clinic diagnosed. 

In the groups studied, VCF was not the original diagnosis after the accident. This is in contrast to the majority of patients typically seen with acute and subacute fractures who very specifically relate the onset of their symptoms to a fall or stressful physical activity. None of the patients under discussion had a previous diagnosis of osteoporosis, although a retrospective review shows that they all were within the expected high risk category (i.e. predominately female gender, age 55 or older, and minor trauma), which should have raised the suspicion of this diagnosis [1-2]. Importantly, 6/10 fractures (Table 1) were at the thoracolumbar junction, which is the most vulnerable area of the spine during seatbelt restrained accidents [4].

In a study by Herzog, et al. (2016), a patient with lumbar spine pathology had 10 MRIs at 10 different spine centers over a three week period [5]. These were interpreted by 10 different radiologists. There were varying radiologic interpretations of all the radiologists and a high prevalence of errors noted on the radiological reports. Regarding vertebral compression fractures, one was noted in this patient by only seven out of 10 radiologists. This demonstrates the importance of treating physicians reviewing the actual imaging studies, not only relying on the reports that could include false-positive and false-negative interpretations. Initially, minor VCFs in patients with osteoporosis may not be visualized and only recognized on follow-up films [1-2]. Most importantly, areas that are not clinically suspected to contain pathology often do and could alter the diagnosis, treatment, and prognosis [3]. Additionally, poor image quality, regardless of type (i.e. plain radiographs, CT, and MRI scans) may lead to false-negatives. This review found the majority of these VCFs to be located at the thoracolumbar junction/lower thoracic spine, an area which can be frequently overlooked or inadequately seen in routine lumbar imaging [3]. There should be a higher index of suspicion if the patient is complaining of persistent pain in a different spinal area after conservative treatment and especially around the thoracolumbar junction [4]. Postmenopausal women, patients on medication such as steroids, or those having malignancy or having undergone chemotherapy should be more carefully screened [5- 6, 7-8].

VCFs may be overlooked with plain radiographs and CT, as the shape of the vertebral body could remain without visual deformity despite a stress fracture being present [2]. With clinical suspicion, an MRI or bone scan should be employed to demonstrate either edematous changes within the vertebral body and/or a fracture line, thereby showing an occult osteoporotic VCF [2- 3]. It is important to differentiate this process from malignant and other benign processes, as the vertebral body visualized on MRI may indicate a disease other than osteoporosis to the radiologist [2, 4, 6-7]. If vertebral augmentation is to occur, performing a bone biopsy as part of the process should at least hopefully rule out a malignant process.

If the patient is not having significant improvement in symptoms, regardless of initial radiologic reports immediately after the injury, follow-up films may reveal a previously undetected fracture [2-3]. Serial imaging needs to be obtained in order to properly visualize a new VCF, a previously undiagnosed VCF, or track a possibly progressive deformity.

## Conclusions

Awareness of the possibility of osteoporotic vertebral compression fractures is the first step in detecting them. This study indicates that in patients with relatively minor trauma, where often the focus is on a simple disc herniation and/or degeneration, there is a small but real risk of overlooking an osteoporotic compression fracture. When necessary, repeat better quality MRI or CT scan, obtain new imaging in another spinal segment due to persistent pain (i.e. the thoracic spine) and perform a bone scan. Patients who are generally over 60 years old, postmenopausal, on corticosteroids or status-post chemotherapy involved in minor trauma are particularly at risk, especially since many have never been diagnosed or treated for osteoporosis. Attention should be focused at the thoracolumbar junction, which is often at the upper edge of lumbar MRIs and/or poorly imaged. This area involving the T12 and L1 vertebrae accounted for 60% of our fractures, but as shown in this study findings of multiple fractures, especially in the thoracic spine, are not unusual.

The delayed recognition of these thoracic and lumbar vertebral compression fractures after minor accident cases could present a major problem. With a delay in diagnosis, the critical time for patients to receive the proper treatments responsible for correcting and preventing further spinal deformity and pain has been severely shortened. 
